# Protocol for a feasibility study, without control group, of a combined treatment for PTSD and difficulties in emotion regulation for patients with substance use disorder

**DOI:** 10.1186/s40814-026-01834-6

**Published:** 2026-05-14

**Authors:** Johanna Vigfusdottir, Edvard Breivik, Erlend Mork, Lars Lien, Håkon Stenmark, Egil Jonsbu

**Affiliations:** 1 Department of Psychiatry, More and Romsdal Hospital Trust, Molde, Norway; 2 Faculty of Medicine and Health Sciences, Norwegian University of Science and Technology, Trondheim, Norway; 3 Division of Mental Health and Addiction, Oslo University Hospital, Oslo, Norway; 4 Faculty of Social and Health Sciences, University of Inland, Elverum, Norway; 5 Regional Center for Violence and Traumatic Stress, St. Olavs Hospital, Trondheim, Norway

**Keywords:** PTSD, SUD, Emotion dysregulation, Dialectical behaviour therapy, Narrative exposure therapy, Feasibility

## Abstract

**Background:**

There are high rates of co-occurring post-traumatic stress disorder (PTSD) among patients in treatment for substance use disorder (SUD). PTSD and SUD should be treated simultaneously, which is rarely the case. The reluctance to offer trauma-focused treatment is partially due to fear of increased risk of dropout. PTSD is related to emotion dysregulation and elevated psychological burden, higher dropout rates and increased risk of relapse. In this project, we plan to assess if it is relevant, feasible, acceptable and safe to add a combination of narrative exposure therapy (NET) and dialectical behaviour therapy for substance use disorder skills training (DBT-SUD Skills) to standard inpatient SUD treatment.

**Methods:**

We will recruit patients from a long-term inpatient SUD treatment centre on the west coast of Norway (*N* approx. = 90). We will assess relevance based on the prevalence of PTSD/Subthreshold-PTSD and traumatic experiences, suicidal behaviour, self-harm, and the severity of difficulties in emotion regulation. We will assess acceptability with treatment participation among patients and the subjective experience of the treatment. We will measure safety with the rate of dropout and destructive behaviour in the treatment period.

**Discussion:**

It is important to develop and evaluate treatment options for this vulnerable patient population, often excluded from clinical research. Strengths include a naturalistic setting and historical data for comparison. Limitations are the absence of a control group and inability to isolate component effects. The results can be used to develop a treatment protocol for combining NET and DBT-skills training to in patient SUD treatment for further development and testing in a multicentre Protocol following SPIRIT 2025 guidelines.

**Trial registration:**

This trial was retrospectively registered at ClinicalTrials.gov on 3 April 2024 (#203,428).

**Supplementary Information:**

The online version contains supplementary material available at 10.1186/s40814-026-01834-6.

## Background

People withSUD are at high risk of developing serious health problems and have high mortality risk [1]; this is costly both for the individual and for our society. There are effective evidence-based treatments available for SUD [2], but 40–70% of patients will relapse into substance abuse within a year of treatment [3]. Patients with SUD are at a high risk for traumatic exposure with up to 95% of SUD patients reporting a history of traumatic exposure [4]. Approximately 36–50% of patients with SUD have comorbid PTSD [5], with a further 33–39% having subthreshold PTSD [6]. Patients with a combination of SUD and PTSD benefit less from standard SUD treatment. They have stronger drug cravings [7], more severe substance use [8], higher risk of SUD treatment dropout and relapse to drug abuse [9–11]. The lack of benefits from standard SUD treatment could be related to their difficulties in emotion regulation and emotional avoidance [11], as substance abuse can be used to avoid difficult emotions [12].

National and international guidelines recommend that SUD and comorbid PTSD should be treated simultaneously and within the same treatment system [13], but that is rarely the case [14, 15]. Trauma-focused exposure therapies are effective to reduce PTSD symptoms and thus over time can reduce the risk of relapse into substance abuse [3]. NET is a trauma-focused therapy developed for people with complex trauma history that have been exposed to persistent and repeated trauma [16]. Patients with comorbid SUD and PTSD have usually experienced multiple types of trauma [17], and NET would therefore be a good treatment fit for this population. Unfortunately, trauma-focused therapies can cause an increase in drop-out from SUD treatment, possibly due to increased distress during treatment [3]. DBT is an evidence-based treatment for Borderline personality disorder [18], and DBT-SUD skills have shown promising results in the treatment for people with co-occurring SUD and emotional dysregulation [19]. In DBT-SUD skills, the focus is to teach the patient how to use effective and safe strategies to manage their emotional reactions, replacing destructive strategies such as substance abuse [18]. SUD is frequently an exclusion criterion in PTSD treatment trials, limiting evidence for integrated approaches [20]. This gap underscores the need for interventions that address both trauma and addiction concurrently. Internal observations and historical data (2014–2016) from our unit indicated high dropout rates (42%) and relapse concerns [21], as 68% relapsed within a year after completed therapy, motivating the augmentation of standard treatment with DBT-SUD and NET. These data are used only as contextual reference, not as formal comparators, due to lack of prior ethics approval for programme audit. By combining treatment interventions focused on reducing PTSD symptoms among traumatised patients in SUD treatment and teaching them methods to regulate one's emotions, one could potentially increase the treatment effect safely without increase in dropout.

As far as we know no studies have combined trauma-focused therapy (NET) and treatment to increase the ability to regulate emotions (DBT-SUD skills) among SUD patients.

This project aims to evaluate whether integrating a combination of NET and DBT-SUD skills into a SUD treatment is feasible by assessing if it is relevant, accepted and safe. Specifically, we hypothesise that the intervention is anticipated to be relevant to the target population, as indicated by baseline assessments of substance use severity, PTSD prevalence, traumatic experiences, suicidal behaviour, and emotion regulation difficulties. Furthermore, the intervention will achieve predefined criteria for recruitment and retention, show high acceptability as reflected in participation rates and positive treatment evaluations, and maintain safety through low rates of treatment dropout, relapse, self-harm, and suicidal behaviour during the study period.

## Methods

### Design

This is a single-arm feasibility study using a pre–post design without a control group. All participants will receive the intervention, and outcomes will be measured at baseline and after completion of the intervention. The primary aim is to assess the feasibility defined as relevance, acceptability and safety, rather than to evaluate effectiveness. The design was chosen because including a concurrent control group within the same unit is not feasible due to high risk of contamination and ethical concerns. Historical data from the unit (2014–2016) [21] on dropout and relapse will serve as a contextual reference for comparison.

### Feasibility outcomes

Feasibility will be evaluated across three predefined domains: relevance, acceptability, and safety. Outcomes within each domain are operationalised as follows:

*Relevance:* The severity of substance use, the prevalence of PTSD/Subthreshold-PTSD, the prevalence of traumatic experiences also as perpetrators, suicidal behaviour and self-harm, and the severity of difficulties in emotion regulation.

*Acceptability:* The percentage of patients that participate in NET and DBT-SUD skills. The dropout rate from the standard treatment, NET and DBT-SUD skills.

*Acceptability:* The percentage of DBT-SUD skills sessions participated in, as well as the percentage of completed DBT homework.

Acceptability: A self-report questionnaire is designed to evaluate the experience of the treatment. Rating how different elements of the treatment are experienced on a 5-point scale. There are also open-ended questions about what is most and least helpful in the treatment. 

*Safety*: The rate of drop-out from treatment, destructive behaviour, such as suicidal behaviour, episodes of drug use and self-harm in the treatment period.

To assess if implementing the intervention is feasible, acceptable, and safe enough for a full multicentre RCT we opted to use a traffic light system to evaluate if the progress criteria are met. “Green” indicates that the progression criteria are met and warrants going to full trial, “amber” indicates that the criteria are partially met and warrants revision is needed before going to full trial, and “red” indicates that the intervention is not feasible, accepted, safe, or that the potential benefits do not warrant going to full trial (see Table 1).

**Table 1:** Criteria for progression from feasibility trial to multicentre RCT

Progression criteria	Measurement	Green	Amber	Red
Relevance	Proportion of participants that have experienced one or more traumatic events	≥ 75%	45–74%	≤ 44%
Relevance	Proportion of participants with PTSD/SubthresholdU-PTSD	≥ 45%	21–44%	≤ 20%
Relevance	Severity of PTSD symptoms measured with PCL	≥ 33	20 - 32	≤ 19
Relevance	Severity of difficulties in emotion regulation measured with DERS	≥ 95	80 - 94	≤ 75
Feasibility, acceptance and safety	Drop-out from treatment	≥ 31%	31–49%	≤ 50%
Safety	Prevalence of suicide behaviour before and while in treatment measured with C-SSRS	Reduction in suicide behaviour compared baseline	Similar suicide behaviour compared to baseline	Increase in suicide behaviour compared to baseline
Safety	Prevalence of self-harm behaviour before and while in treatment measured with DSHI	Reduction in Self. Harm behaviour compared baseline	Similar self-harm behaviour compared to baseline	Increase in self-harm behaviour compared to baseline
Acceptance	Proportion of participants that accepted participation in DBT- skills	≥ 65%	45–64%	≤ 44%
Acceptance	Proportion of participants that completed DBT-SUD skills	≥ 60%	40–59%	≤ 39%
Acceptance	Proportion of DBT-skills sessions participated in	≥ 75%	41% - 75%	≤ 40%
Acceptance	Proportion of participants that accepted participation in NET	≥ 70%	50–69%	≤ 50%
Acceptance	Proportion of participants that completed NET	≥ 60%	41–59%	≤ 40%
Feasibility and acceptance	Subjected experience of treatment participants	≥ 80% of participants strongly agree or agree the intervention is instructive, useful, meaningful, helpful, feasible, and relevant, and ≥ 80% of participants disagree or strongly disagree that the intervention is too difficult	40–79% of participants agree or strongly agree that the intervention is instructive, useful, meaningful, helpful, feasible, and relevant, and 40–79% of participants disagree or strongly disagree that the intervention is too difficult	≤ 39% of participants agree or strongly agree that the intervention is instructive, useful, meaningful, helpful, feasible, and relevant, and ≤ 39% of participants disagree or strongly disagree that the intervention is too difficult
Feasibility and acceptance	Subjected experience of treatment staff	≥ 80% of participants strongly agree or agree the intervention is instructive, useful, meaningful, helpful, feasible, and relevant, and ≥ 80% of participants disagree or strongly disagree that the intervention is too difficult	40–79% of participants agree or strongly agree that the intervention is instructive, useful, meaningful, helpful, feasible, and relevant, and 40–79% of participants disagree or strongly disagree that the intervention is too difficult	≤ 39% of participants agree or strongly agree that the intervention is instructive, useful, meaningful, helpful, feasible, and relevant, and ≤ 39% of participants disagree or strongly disagree that the intervention is too difficult

For further information on the rational for each criterion, se Attachment 1

#### Recruitment of participants

All patients admitted to the inpatient programme at Molde Treatment Center (MBS) from May 2021 to May 2024 will be invited to participate (*N* approx. = 90). Recruitment is continuous and consecutive in the recruitment period. All participants will participate in the standard treatment at MBS. Those who meet the inclusion criteria for further intervention will also be invited to participate in NET and/or DBT-SUD skills (*N* approx. = 75). Interventions begin 1–2 months post-admission to allow stabilisation. DBT-SUD groups will be formed on a rotating basis with 6–8 participants per cohort.

**Fig. 1:**
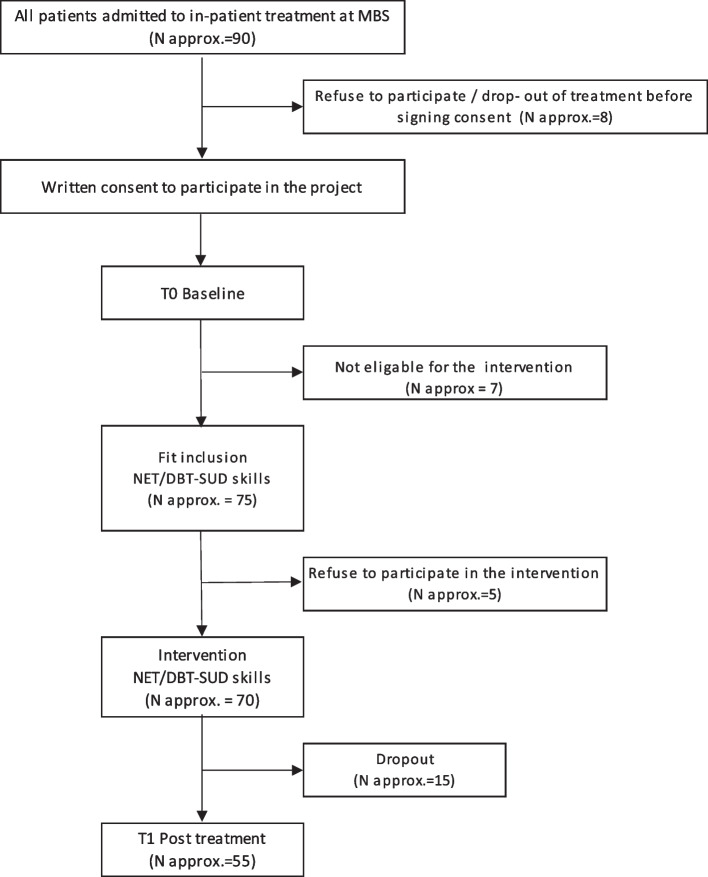
Participant flow chart

Recruitment and follow-up plan is shown with patient flow chart in Fig. 1.

*Sample size:* The target sample size for this study is 75 participants. As this is a feasibility study, the sample size is not powered to detect treatment effects, but is intended to give estimates of recruitment, retention, acceptability, and safety parameters, as well as preliminary estimates of outcome variability to inform future definitive trial.

Guidelines for feasibility studies suggest that sample sizes between 24 and 70 participants are generally sufficient to estimate key feasibility parameters with acceptable precision, depending on study aims and outcome variability [22, 23]. Larger samples can be needed when evaluating multiple feasibility outcomes and when recruitment occurs in routine clinical settings [22, 23].

The unit admits approximately 20–30 patients annually, and recruitment will take place over a three-year period, providing an estimated pool of 60–90 eligible patients. Historical data from the unit indicate an acceptance rate of around 75%, with retention rates comparable to those observed in similar feasibility studies [21]. Based on these assumptions, inviting approximately 90 patients is expected to yield 75 participants, which is considered sufficient to meet the objectives of the feasibility assessment [22, 23].

### Data collection

**Fig. 2:**
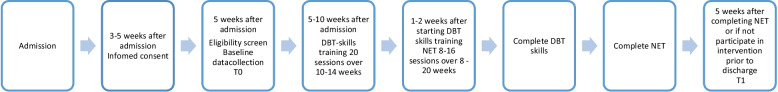
The schedule and timeline for recruitment, data collection and the intervention

We will collect data: Five weeks after admission (T0), four weeks after completion of NET (T1). The schedule and timeline for the enrolment, data collection and intervention are shown in Fig. 2 and an overview of measurements at each data collection point is shown in Table 2.

**Table 2:**
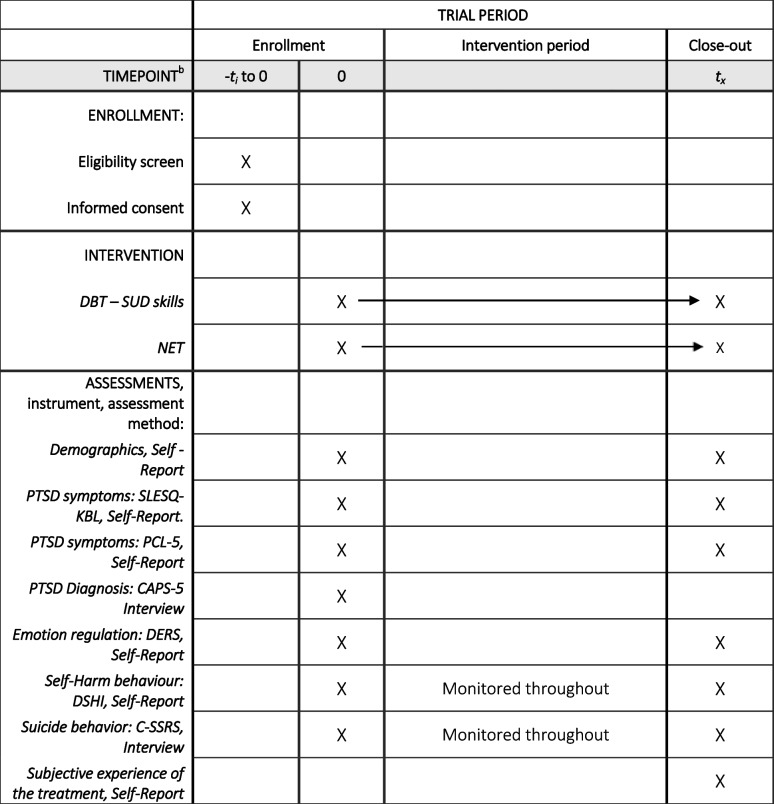
Participant timeline: Schedule of enrolment, interventions, and assessments

^*^In accordance with the SPIRIT 2025 guidelines [47]

General inclusion criteria for the study:

1. Fit general inclusion criteria for the in-patient programme.
2. Speak Scandinavian (Norwegian, Danish and/or Swedish).
3. Be willing to sign the written informed consent.

General exclusion criteria for the study:

1. Have a clinically significant low cognitive- and/or linguistic functioning that hinders the patient in understanding and answering the questions on the self-report instruments.

#### Inclusion criteria for NET intervention

1. Experience of an aversive event that fits criteria A for PTSD as defined by Diagnostic and Statistical Manual of Mental Disorders-V (DSM-V) [24].
2. Experience symptoms of PTSD as defined by the DSM-V [24], or subthreshold PTSD [25].
  - Subthreshold PTSD is defined as having experienced a traumatic event (Criteria A), meeting Criteria B (re-experiencing symptoms), Criteria E (1-month symptom duration), and Criteria F (significant distress or functioning impairment) and either Criteria C (avoidance or numbing symptoms) or Criteria D (hyper arousal symptoms).

#### Inclusion criteria for DBT intervention

1. A history of pervasive difficulties in understanding and managing emotions as evaluated by an assigned DBT therapist.
2. Manage to commit to participating in the DBT-skills training.

#### Exclusion criteria for NET and/or DBT-SUD skills

1. Being actively psychotic.
2. Have a body mass index (BMI) under 17.
3. Severe dissociation.
4. An ongoing traumatic contact with the perpetrator.

### The intervention

The average treatment period is 6 to 9 months; therefore, the intervention period will be from May 2021 to approximately October 2024. Patients start NET and DBT-SUD skills training one to two months after admission.

#### Molde Treatment Centre (MBS)

The institution is an in-patient, drug rehabilitation centre with room for 15 patients. Before admission, the patient usually has already been through the detox and abstinence phase. Patients are expected to be absent from substance use while in treatment at the facility. Standard treatment at MBS consists of a version of the therapeutic community (TC), a purposefully designed miniature drug-free social environment or residential treatment setting with clear rules to promote social and psychological change [26], family therapy [27], and CBT for SUD, that uses cognitive behavioural principles to manage drug addiction. CBT-SUD is delivered both individually and in-group settings [28].

#### Narrative exposure therapy (NET)

Narrative exposure therapy (NET) is a treatment for trauma disorders, particularly for individuals suffering from complex and multiple trauma [16]. With the guidance of the therapist, a patient establishes a chronological narrative of his or her life, concentrating mainly on their traumatic experiences represented with stones, but also incorporating some positive events represented with flowers [29]. For patients who have themselves been perpetrators, we will add the narrative of violent/sexual offences represented by sticks as characterised in the further developed version of FORNET [30]. The therapy combines mapping, accepting, and exposure to one’s experiences. In the last session, a documented autobiographic narrative created by the therapist is presented to the patient.

The NET intervention consists of 10–18; 90-min sessions with a NET therapist once or twice a week for 5–10 weeks.

- One session of introduction and psychoeducation on PTSD-SUD and NET treatment.
- One–2 sessions laying the lifeline.
- Three–16 sessions with working and writing the narrative, with the narrative repeated at the start of each session.
- One session where the complete narration is read and handed out in the last session.

The patient often chooses to use the narration in family therapy sessions and share in treatment with a group of staff and fellow patients they themselves select.

#### NET training and quality control

NET will be delivered by qualified health personnel, including psychiatrists, psychologists, or other licensed clinicians, who have received specific training in the intervention. Training consists of a three-day workshop organised by the Regional Centre for Violence, Traumatic Stress and Suicide Prevention (RVTS), followed by structured follow-up and monthly supervision sessions to support fidelity and quality control. The training programme provides theoretical instruction on the principles of NET, practical exercises in constructing lifelines, and supervised practice sessions. These measures aim to ensure that therapists deliver NET in accordance with standardised protocols and maintain adherence throughout the trial.

#### DBT-SUD skills

DBT-SUD skills is a group skills training component of dialectical behaviour therapy, a comprehensive treatment focused on extensive difficulties in emotion regulation [18]. In this project, we will use a combination of the trans-diagnostic skill-training model by Neacsiu [12, 31] with the addition of SUD-specific skills. Every patient participating in the DBT skills training is assigned a DBT-trained therapist.

The DBT-SUD skills intervention consists of:

- Two–3 sessions with a DBT therapist focusing on treatment orientation—mapping the patients' goals, obstacles, and resources, DBT-hierarchy, and commitment.
- Skills training—20, 2-h sessions—twice a week over a period of 10 to 11 weeks. The skills consist of Mindfulness, Distress Tolerance, Interpersonal Effectiveness, Emotion regulation, and SUD-specific skills.
- Chain analysis in case of an episode of substance use or other dangerous destructive behaviour.
- One summary session with a focus on goals reached and troubleshooting.
- Support from DBT skills-oriented staff and environment.

Two DBT therapists lead the group, and each group consists of approx. 6 - 8 participants.

#### DBT training and quality control

The DBT-SUD skills training will be delivered by qualified health professionals, including psychiatrists, psychologists, or other licensed clinicians, who have completed comprehensive DBT training. This training consists of two intensive modules delivered by certified trainers. The first module consists of a 5-day workshop and 6 months follow-up that focuses on foundational DBT principles, including behavioural analysis, mindfulness, and skills training, and introduces the theoretical framework and core strategies for individual therapy and skills groups. The second module consists of a 4-day workshop that emphasises advanced application, team consultation, and troubleshooting complex cases, consolidating learning from the initial module, and ensuring fidelity to the DBT model through supervised practice and case discussions. Following completion of both modules, therapists undergo follow-up evaluation to confirm competence. To maintain treatment integrity, external supervision will occur every 2 months, and weekly consultation team meetings will provide ongoing supervision and quality control throughout the trial.

#### Assessment of harmful behaviours

In this study, harmful behaviours are defined as any adverse events or behaviours indicative of increased risk or deterioration in participants’ well-being during the trial. Specifically, harmful behaviours encompass relapse (return to substance use), treatment dropout, self-harm behaviours, and suicidal ideation or attempts. These outcomes will be systematically assessed at baseline and throughout the intervention period using validated instruments: the Columbia-Suicide Severity Rating Scale (C-SSRS) for suicidal ideation and behaviour, the Deliberate Self-Harm Inventory (DSHI) for self-harm, and clinical records for relapse and treatment discontinuation. Monitoring will occur in response to any clinical concerns raised by staff.

#### Management of adverse events

Upon identification of harmful behaviour, standard clinical protocols at the treatment facility will be implemented. These procedures include comprehensive risk assessment and chain analysis of the problematic behaviour, safety planning and crisis intervention, reinforcement of treatment engagement strategies, and increased monitoring and supervision as clinically indicated. All adverse events will be documented in the trial database and reviewed by the clinical team. Serious adverse events, such as suicide attempts or severe self-harm, will be reported in accordance with institutional policies and regulatory requirements.

### Measurement

#### Demographics

Basic demographic information from the participants is registered at admission. This includes age, gender, nationality, immigration status, level of education, employment/support status, living situation, marital status, children and custodianship, medical status, and legal status e.g. if the treatment is part of a criminal sentence. Questions regarding smoking or non-smoking and substance use cravings are also included. Information about ICD-10 drug diagnoses (F10 - 19, indicating substance used) and previous SUD inpatient stay, previous inpatient stay (yes/no), onset of substance use, poly drug use (yes/no), and injecting drug use (yes/no) will be obtained from medical records.

#### Relevance

##### Traumatic experiences and PTSD symptoms

*Stressful Life Events Screening Questionnaire-Revised (SLESQ)*is a self-report instrument designed to map and assess 15 potentially traumatic experiences [32]. For this project, items asking about experiences where the participant caused potentially traumatic experiences to others are added. This is to identify violent perpetrators. Participants with one or more yes answer PTSD Checklist for DSM-5.

*PTSD Checklist for DSM-5 (PCL-5)*is a self-report instrument developed for quick screening of PTSD symptoms. Sum scores range from 0 to 80, with a score over 33 indicating the presence of PTSD [33].

*Clinician-Administered PTSD Scale for DSM-5 (CAPS-5)*is a 30-item structured interview used to make current (past month) diagnosis of PTSD [34]. Frequency and intensity rating is summed to create an overall PTSD symptom severity score and is used to generate a categorical diagnosis (PTSD, Subthreshold-PTSD, non-PTSD). In this project, we will both use the categorical diagnosis to measure the prevalence of PTSD and Subthreshold-PTSD [34].

##### Emotion regulation

*Difficulties in Emotion Regulation Scale (DERS*) is a self-report instrument consisting of 36 items meant to measure difficulties in emotion regulation, higher scores ranging from 36 to 144 indicate more significant difficulties in emotion regulation [35]. We will use the cut score of 97 to identify severe difficulties in emotion regulation [31, 36].

#### Safety

##### Aversive behaviours: self-harm, suicide behaviour

*The Deliberate Self-Harm Inventory (DSHI*). As a part of this project, we did a translation and assessed the psychometric properties of DSHI in a Norwegian population [37]. DSHI is a 17-item behaviourally based, self-report instrument to assess deliberate self-harm [38]. In this project, we create a continuous variable on frequency of self-harm behaviour and a dichotomous variable on presence of self-harm (Yes/No).

*Columbia-suicide severity rating scale (C-SSRS)*is a suicidal ideation and behaviour-rating interview created to evaluate suicide risk. The interview consists of 10 categories with binary responses (yes/no) to indicate a presence or absence of the behaviour. The outcome of the C-SSRS is a numerical score obtained from the categories [39].

##### Dropout from treatment

Dropout is registered, and dichotomous variables are created (yes/no). In case of dropout, time from admission to dropout is registered.

##### Acceptability

To measure acceptability, we register the proportion of participants that accepted participation in DBT-skills training and NET (separately), the proportion of participants that completed NET, and the proportion of DBT-skills training sessions they participated in.

For the purpose of this study, a self-rapport questionnaire was designed to evaluate the experience of DBT-Skills and NET. Consisting of seven questions on the experience of each intervention (DBT and NET). Rating on a 5-point Likert scale how different elements of the treatment are experienced on a 5-point scale (Instructive, Useful, Too difficult, Meaningful, Helpful, Feasible, Relevant). There are also open-ended questions about what is most useful and least helpful in the treatment (Attachment 2).

### Statistical analysis

The primary analyses will focus on feasibility outcomes, specifically indicators of relevance, acceptability, and safety. Recruitment rates, intervention uptake, session attendance, completion rates, and the frequency of adverse events (dropout, relapse, self-harm, suicide behaviour) will be summarised using proportions and percentages, with 95% confidence intervals where appropriate.

Descriptive statistics will also be provided for demographic and psychological factors to contextualise feasibility outcomes. Continuous variables will be reported in terms of mean or median value with appropriate measure on spread (standard deviation or quartiles), depending on distributional properties. The distribution of categorical factors will be reported in terms of proportions and percentages.

### Ethics

Attending study is voluntary and requires informed consent. Refusing participation in the study will not affect patient’s access to treatment. The interventions in the study will not hinder any other well-documented treatment at MBS. The participation is time-consuming and demanding for the patient and could temporarily affect the patient's ability to participate in social interaction in the therapeutic community. Answering questions about traumatic experiences can potentially cause discomfort. A therapist and other staff at the clinic will be there to help the patient regulate possible transitory increase in PTSD symptoms. There are a number of potential benefits for participants in this project such as access to treatment that can lead to symptom reduction, less suffering, greater quality of life and reduction in relapse into drug abuse. Benefits from participation are supposed to outweigh potential discomfort. Two trained patient representatives with lived experience have and will participate in the trial steering committee to provide input on participant burden, protocol, the design of the intervention, acceptability, and communication materials.

### Data protection, management and storage

Self-report questionnaires will be collected in paper format by the primary researchers at the institution, and structured interviews will be conducted by trained personnel using standardised protocols. All data will be stored according to established protocols on data protection of More & Romsdal Health Trust. The information will be stored in a de-identified manner. Completed questionnaire packages will be stored in a locked filing cabinet and identified only by a research ID number. The identification key linking research IDs to personal identifiers will be kept in a separate locked cabinet, accessible exclusively to the principal investigators. To ensure data quality, double data entry and periodic cross-checks will be performed, with any discrepancies resolved by the research team. Access to the identification key will be restricted to the main researchers, and all actions will be logged for audit purposes. Data will be retained for up to five years following project completion, after which all physical and electronic records will be securely destroyed in accordance with institutional policies and GDPR requirements. All statistical analysis and use of data collected in this project will be done on a group level.

## Discussion

This is a feasibility study, without control group, of a combined treatment for PTSD and difficulties in emotion regulation for patients with substance use disorder. There are high rates of PTSD among inpatients with SUD [5, 41], and a high risk of relapse after treatment [9–11]. By identifying subgroups of patients with SUD, comorbid PTSD and difficulties in emotion regulation, and developing treatment targeting specifically those areas, one could potentially decrease the suffering of a patient population often excluded from PTSD treatment trials and possibly decrease relapse to substance abuse [3].

This study aims to develop and check the relevance, acceptability and safety of adding to standard SUD inpatient treatment a combination of trauma-focused therapy (NET) and treatment for difficulties in emotion regulation (DBT-SUD-Skills). Specifically, we hypothesise that the intervention is anticipated to be relevant to the target population, as indicated by baseline assessments of substance use severity, PTSD prevalence, traumatic experiences, suicidal behaviour, and emotion regulation difficulties. Furthermore, the intervention is expected to achieve predefined criteria for recruitment and retention, show high acceptability as reflected in participation rates and positive treatment evaluations, and maintain safety through low rates of treatment dropout, relapse, self-harm, and suicidal behaviour during the study period.

### Strength and limitations

#### Strength

This is a feasibility study in a quite large sample in naturalistic long-term treatment, making the results generalisable. We have historical data from prior study in the institution on dropout to use as a preliminary reference for comparison. The result of the study is expected to give relevant information in a population that is often excluded from studies [20].

#### Limitations

With a lack of a randomised control group, one cannot measure the effect of the intervention. The combined introduction of DBT-SUD and NET limits attribution of effects to individual components. A factorial design (TAU vs. TAU + DBT-SUD vs. TAU + NET vs. TAU + NET + DBT-SUD) would provide stronger evidence. Due to the structure of the treatment centre, which involves long-term inpatient treatment, it is not feasible to include a concurrent comparison group within the same centre. The risk of contamination between participants is considered too high for a valid comparison. Furthermore, denying a patient access to an intervention that other patients in the same setting receive could be ethically problematic and may lead to frustration among participants. To include a proper comparison group, a multicentre study would be required. Such a design demands substantial financial and logistical resources. Therefore, it is essential first to establish whether the intervention is relevant, accepted and safe, and relevant in this setting. Historical data on dropout and relapse collected at the unit between 2014 and 2016 [21] will serve as a preliminary reference for comparison. This limitation will be acknowledged, and future trials may adopt a multi-arm design.

In effort to increase the validity and quality of the study, we have followed the Standard Protocol Items: Recommendations for Interventional Trials guidelines from 2025 [41] as can be seen in the SPIRIT 2025 checklist in Attachment 3.

## Trial status

We are closed for recruitment, with the last participant being included at the start of May 2024. The last participant completed treatment participation at the end of October 2024.

## Future research

If the intervention fits the progression criteria and proves to be relevant, feasible, acceptable and safe, and of potential benefit for the patients, the future aim is to conduct a multi-arm multicentre randomised control trial (RCT) on the effect of combined NET and DBT-SUD skills integrated into SUD treatment.

## Supplementary Information

Supplementary Material 1.

Supplementary Material 2.

Supplementary Material 3.

## Data Availability

The data can be made available on request. Clinical deidentified data, data dictionary and statistical code will be available to approved applicants after the publication of the main findings within five years of the trial finishing. Clinical data access requests are to be sent to the corresponding author: Johanna Vigfusdottir.

## Abbreviations

PTSD: Post-traumatic stress disorder
SUD: Substance use disorder
NET: Narrative exposure therapy
DBT-SUD skills: Dialectical behaviour therapy for substance use disorder skills training
SUB-PTSD: Subthreshold post-traumatic stress disorder
RCT: Randomised controlled trial
MBS: Molde Treatment Center
T0: First point of evaluation 5 weeks after admission
T1: Second point of evaluation, four weeks after completion of NET
DSM-5: Diagnostic and Statistical Manual of Mental Disorders, fifth revision
BMI: Body mass index
ICD-10: The International Classification of Diseases, tenth revision
AUDIT: Alcohol Use Disorders Identification Test
DUDIT: Drug Use Disorder Identification Test
SLESQ: Stressful Life Events Screening Questionnaire-Revised
PCL-5: PTSD Checklist for DSM-5
CAPS-5: Clinician-Administered PTSD Scale for DSM-5
DERS: Difficulties in Emotion Regulation Scale
DSHI: The Deliberate Self-Harm Inventory
C-SSRS: Columbia-Suicide Severity Rating Scale
