# Supplementary material for: Protocol for a feasibility study, without control group, of a combined treatment for PTSD and difficulties in emotion regulation for patients with substance use disorder
Source: Pilot Feasibility Stud. 2026 May 14;12:97. doi: 10.1186/s40814-026-01834-6 (PMC13343655; doi:10.1186/s40814-026-01834-6)
Supplement: Supplementary file 1 — Supplementary Material 1. [file 40814_2026_1834_MOESM1_ESM.docx]

**Attachment 1**

**Rational for progress criteria from feasibility studie to a multicentre RCT**

**Relevance - Prevalence of exposure to traumatic events:** Patients with SUD are at a high risk for traumatic exposure with up to 95% of SUD patients reporting a history of traumatic exposure. ^41^ Exposure rate of 75% having been exposed to one or more traumatic event would indicate high relevance (green), a exposure rate of 45% - 74% indicates some relevance (amber), and exposure rate of 44% or lower could indicate low relevance (red).

**Relevance - Prevalence of PTSD:** Approximately 36-50% of patients with SUD experience comorbid PTSD.^5^ Prevalence rate of PTSD of 45% indicates high relevance (green), Prevalence of 21% - 44% indicates some relevance (amber), a prevalence rate of 20% or lower would indicate low relevance (red).

**Relevance - Severity of PTSD symptoms measured with PCL:** Sum score of 31-33 is indicative of probable PTSD.^33^ A collective mean is set at the clinical cut-off (green). Mean score of 31 or higher indicates high relevance (green), a score of 20 – 30 indicates some possible relevance (amber), a score of 19 or lower would indicate low PTSD symptom severity and low relevance (red).

**Relevance - severity of difficulties in emotion regulation measured with DERS:** The mean score of 97 seems to indicate severe difficulties in emotion regulation.^31, 36^ The mean score for the normal population is 75,^42^ indicating non-excessive difficulties in emotion regulation. Mean score of 95 or higher indicates high relevance (green), a score 76 – 94 indicates some relevance (amber), and score of 75 or less would indicate low relevance (red).

**Acceptability, feasibility and safety –Dropout rate:** The dropout rate from the institution for TAU was 31%.^21^ No increase in drop-out the intervention would be assumed safe (green), some increase in drop-out 32 – 44% would be within safety but worth assessing (amber), but great increase in dropout 45% or over we would have to re-evaluate the safety of the treatment (red).

**Safety - Prevalance of suicide behaviour before and while in treatment measured with C-SSRS:** Almost one tenth (9,3 %) of persons committing suicide in Norway have been in contact with addiction related health care, in the year prior to committing suicide. Combined prevalence for committed suicide while hospitalized or on leave from, either in substance use treatment or psychiatric treatment is approximately 7%.^43^ We collect baseline data on suicide behavior, both lifetime and in the last 6 months. Any increase in suicide attempts from baseline data would be alarming. Reduction in suicide behavior compared to baseline is safe (green), the same rates of suicide behavior are within safety (amber), any increase in rates of suicide behaviour is alarming (red).

**Safety - Prevalance of Self-harm behaviour before and while in treatment measured with DERS:** We collect baseline data on the self-harm behaviour, both lifetime and in the last 6 months. Any increase in Self-harm from baseline data would be alarming. Reduction in self-harm behavior compared to baseline is safe (green); the same rates of self-harm behavior are within safety (amber); any increase in rates of suicide behaviour is alarming (red).

**Acceptance - Proportion of participants that accept participation in DBT- skills.** In a homogenous sample of patients with substance use disorder, one can assume that not all treatment interventions are experienced as relevant. DBT skills training focuses on emotion regulation and is dependent on the patient’s own motivation or experienced need for emotion regulation (Linehan, 2013). Prior research has shown up to 43% refusal rate in participating in DBT skill training among patients in SUD treatment.^44^ If 65% of the participants agree to participate in DBT Skills training the intervention is assumed accepted (green), 45% - 64% agreement to participate it is assumed to be somewhat accepted (amber), and 44% or lower it will be at risk for not being accepted (red).

**Acceptance - Proportion of DBT skills sessions participated in:** To be able to use and benefit from the use of relevant skills taught in DBT skills training requires active participation in training. It is usual to set criteria for skills completion, to attending rate of 75% of sessions.^44^ Attending 75% of the sessions or more is a criterion for the acceptance of the intervention (green), attending 50% - 74% of the sessions it is assumed the intervention is partially accepted (amber), attending 49% of the skills sessions there is a risk of the intervention not being accepted (red).

**Acceptance - Proportion of participants that completed DBT-SUD skills:** Completion is a strong indicator of intervention acceptability because it reflects sustained engagement and perceived relevance over time. ^12, 44^ Prior studies in SUD populations report substantial attrition in DBT skills programs, with completion rates varying widely (often below 60%) due to motivational and contextual factors.^44^ Based on feasibility literature and pragmatic thresholds used in pilot studies,^4, 45^ we propose the following criteria: Green (Accepted): ≥ 60% of participants complete the DBT-SUD skills module, Amber (Partially Accepted): 40% – 59% completion, Red (At Risk): ≤ 39% completion

**Acceptance - Proportion of participants that accepted participation in NET:** One of the hinder in implementing trauma-focused therapies in combination with SUD treatment is the fear that the patient is not ready nor willing to participate in trauma exposure. The proportion of participation in NET is therefore an important measure of acceptance. ^46^ If 70% of the participants agree to participate in NET, the intervention is assumed to be accepted (green), 50% - 69% agreement to participate in it is assumed to be somewhat accepted (amber), and 49% or lower it will be at risk for not being accepted (red).

**Acceptance - Proportion of participants that completed NET:** One of the hinders in implementing trauma-focused therapies in combination with SUD treatment is the fear of dropout from treatment.^46^ There is some evidence that supports the theory of increased drop-out rates with trauma-focused therapies. This also seems to apply to integrated PTSD and SUD treatment. ^3^ If 75% of the participants that start NET complete the treatment, the intervention is assumed to be accepted (green), 50% - 74% complete NET it is assumed to be somewhat accepted (amber), and 49% or lower it will be at risk for not being accepted (red).

**Feasibility and acceptance - Subjective experience of treatment:** If 80% of participants strongly agree or agree the intervention is instructive, useful, meaningful, helpful, feasible, and relevant, and 80% of participants disagree or strongly disagree that the intervention is too difficult it is assumed to be feasible and accepted (green). If 40% - 79% of participants agree or strongly agree that the intervention is instructive, useful, meaningful, helpful, feasible, and relevant, and 40% - 79% of participants disagree or strongly disagree that the intervention is too difficult it is assumed somewhat accepted (amber). But if 39% or less agree or disagree on the terms, there is a risk that the intervention is not feasible nor accepted (red).
