# Supplementary material for: Protocol for a feasibility study, without control group, of a combined treatment for PTSD and difficulties in emotion regulation for patients with substance use disorder
Source: Pilot Feasibility Stud. 2026 May 14;12:97. doi: 10.1186/s40814-026-01834-6 (PMC13343655; doi:10.1186/s40814-026-01834-6)
Supplement: Supplementary file 2 — Supplementary Material 2. [file 40814_2026_1834_MOESM2_ESM.docx]

**Attachment 2**

Translated version

| Your experience of DBT-SUD skills training and NET, patient. | | | | | |
| --- | --- | --- | --- | --- | --- |
|  | Strongly agree | Agree | Neutral | Disagree | Strongly disagree |
| **How did you experience DBT-SUD skills training?** |  |  |  |  |  |
| Instructive | 1 | 2 | 3 | 4 | 5 |
| Useful for my problems | 1 | 2 | 3 | 4 | 5 |
| Too difficult | 1 | 2 | 3 | 4 | 5 |
| Meaningful | 1 | 2 | 3 | 4 | 5 |
| Helpful | 1 | 2 | 3 | 4 | 5 |
| Feasible | 1 | 2 | 3 | 4 | 5 |
| Relevant for my problems | 1 | 2 | 3 | 4 | 5 |
| **How did you experience NET?** |  |  |  |  |  |
| Instructive | 1 | 2 | 3 | 4 | 5 |
| Useful for my problems | 1 | 2 | 3 | 4 | 5 |
| Too difficult | 1 | 2 | 3 | 4 | 5 |
| Meaningful | 1 | 2 | 3 | 4 | 5 |
| Helpful | 1 | 2 | 3 | 4 | 5 |
| Feasible | 1 | 2 | 3 | 4 | 5 |
| Relevant for my problems | 1 | 2 | 3 | 4 | 5 |
|  |  |  |  |  |  |
| What has been most helpful in the treatment? | |  |  |  |  |
|  | |  |  |  |  |
|  |  |  |  |  |  |
|  |  |  |  |  |  |
|  |  |  |  |  |  |
| What has been least helpful in the treatment? | | |  |  |  |
|  |  |  |  |  |  |
|  |  |  |  |  |  |
|  |  |  |  |  |  |
|  |  |  |  |  |  |
