# Supplementary material for: Protocol for a feasibility study, without control group, of a combined treatment for PTSD and difficulties in emotion regulation for patients with substance use disorder
Source: Pilot Feasibility Stud. 2026 May 14;12:97. doi: 10.1186/s40814-026-01834-6 (PMC13343655; doi:10.1186/s40814-026-01834-6)
Supplement: Supplementary file 3 — Supplementary Material 3. [file 40814_2026_1834_MOESM3_ESM.docx]

**Attachment 3.** SPIRIT 2025 checklist of items to address in a randomised trial protocol.^41^

| Section/topic | No | SPIRIT 2025 checklist item description |
| --- | --- | --- |
| **Administrative information** | | |
| Title and structured summary | 1a | Protocol for a feasibility study, without control group, of a combined treatment for PTSD and difficulties in emotion regulation for in-patients with substance use disorder. |
|  | 1b | Manuscript: Figure 2 |
| Protocol version | 2 | Protocol version 4.0 from 15.12.2025, replacing version 3.0 from 03.05.2024 adding more detailed information in accordance with the SPIRIT 2025 guidelines. 3.0 version replaced the 2.0 version from 15.03.24 (Published at Clinicaltrials.gov 02.04.2024), as the 2.0 version was to broad, including an observational study and variables that were not as relevant for the feasibility of the intervention. The original version 1.0 was written on 04.04.2021 but was not published (outside the application to the regional ethics committee). The 2.0 version replaced the 1.0 version of the protocol, improving the structure of the protocol, and adding details about methods, instruments, and analysis. |
| Roles and responsibilities | 3a | Johanna Vigfusdottir^1,^ Clinical psychologist, Phd student and main researcher, implementation DBT, quality control and data collection, data analysis and design.  Edvard Breivik^1^, Psychiatrist, PhD student, research group, implementation of NET, quality control and data collection and design.  Erlend Mork^2^, PhD clinical psychologist, study chair research group, quality control, advisor  Lars Lien^3^ Professor, MD, study chair research group, quality control, advisor.  Håkon Stenmark^4^ PhD, clinical psychiatrist, study chair research group, quality control, advisor  Study director and trial sponsor: Egil Jonsbu^1.^  ^1^ More and Romsdal Hospital Trust and Norwegian University of Science and Technology ^2^ Oslo University Hospital ^3^ National Competence Center for Co-Occurring Addictive and Psychiatric Disorders ^4^ Regional Center for Violence and Traumatic Stress and St. Olavs Hospital  Manuscript: Declarations – Author contribution: p. 14, line 25 - 30  All |
|  | 3b | The trial sponsor:  Møre and Romsdal Hospital Trust  Contact: Egil Jonbu, egil.jonsbu@helse-mr.no |
|  | 3c | More and Romsdal Hospital Trust (HMRT) is responsible for ensuring the study is designed and conducted in accordance with research guidelines. This includes overseeing the development of the research protocol, ethical considerations, as well as securing regulatory and ethics approvals. HMRT provides financial and logistical resources, oversees trial operations, monitors data quality and participant safety, and ensures compliance with Good Clinical Practice and relevant regulations. Additionally, the HMRT is accountable for transparent trial registration, timely reporting of results, and adherence to open-science principles, including patient and public involvement throughout the research process. |
|  | 3d | The data management committee (Johanna Vigfusdottir, Edvard Breivik, statistics: Tor Åge Myklebust and Egil Jonsbu) will ensure data quality, double data entry and periodic cross-checks will be performed, resolve discrepancies and be responsible for data analysis. Johanna Vigfusdottir, Edvard Breivik and Egil Jonsbu are responsible for governance, quality control, and supervision throughout the trial. They maintain accountability, and coordinate with the sponsor, who holds ultimate oversight. The group supports the establishment and monitoring of systems for data integrity, protocol adherence, and participant safety. They manage version control and amendments, ensuring all changes are documented and approved. Feasibility-specific supervision includes tracking recruitment, retention, and predefined stopping criteria to assess trial viability. Ethical compliance and clear processes for adverse event reporting and open science practices, including data-sharing plans. The rest of the research committee (Erlend Mork, Lars Lien and Håkon Stenmark) contributes with support and guidance in that work. Johanna Vigfusdottir and Edvard Breivik are responsible for the day-to-day operations and implementation of the treatment interventions. |
| **Open science** | | |
| Trial registration | 4 | Registered at clinicaltrials.gov on the 03.04.2024, [Study Details \| NCT06345053 \| Presence of PTSD and Emotion Dysregulation Among Inpatients With Substance Use Disorder \| ClinicalTrials.gov](https://clinicaltrials.gov/study/NCT06345053?cond=PTSD&term=Substance%20Use%20Disorder&intr=DBT&rank=2) |
| Protocol and statistical analysis plan | 5 | At clinicaltrials.gov. [Study Details \| NCT06345053 \| Presence of PTSD and Emotion Dysregulation Among Inpatients With Substance Use Disorder \| ClinicalTrials.gov](https://clinicaltrials.gov/study/NCT06345053?cond=PTSD&term=Substance%20Use%20Disorder&intr=DBT&rank=2#more-information) |
| Data sharing | 6 | Manuscript: Declarations - Availability of data end material |
| Funding and conflicts of interest | 7a | Norwegian University of Science and Technology.  Helse More and Romsdal Hospital trust |
|  | 7b | No conflict of interest to declare |
| Dissemination policy | 8 | Publications in peer reviewed journals, and conferences both within the research and clinically relevant community such as addiction and trauma, but also within the patient populations and user organizations. |
| **Introduction** | | |
| Background and rationale | 9a | Manuscript: Introduction |
|  | 9b | Manuscript: Discussion – Strengths and limitations -Limitations. |
| Objectives | 10 | Manuscript: Methods – Design, Methods – The intervention – Assessment of harmful behaviors – Management and Table 1. |
| **Methods: Patient and public involvement, trial design** | | |
| Patient and public involvement | 11 | Manuscript: Methods - Ethics |
| Trial design | 12 | Manuscript: Methods - Design |
| **Methods: Participants, interventions, and outcomes** | | |
| Trial setting | 13 | Manuscript: Methods – Intervention – Molde Treatment Centre |
| Eligibility criteria | 14a | Eligibility criteria for participants  Manuscript: Methods - General inclusion criteria - exclusion criteria, inclusion for DBT intervention, Inclusion for NET intervention |
|  | 14b | Manuscript: Method - Intervention Narrative exposure therapy and Dialectical behavior therapy: |
| Intervention and comparator | 15a | Manuscript: Methods - Intervention - Narrative exposure therapy – NET training and quality control.  Manuscript: Methods – Intervention – Dialectical behavior therapy – DBT training and quality control. |
|  | 15b | Manuscript: Methods – The intervention - Assessment of harmful behaviors  Criteria for the intervention to be discontinued or modified for individual participants' safety thresholds are breached are outlined in Table 1.  Participants may also request discontinuation at any time, and clinical judgment will override protocol if participant safety is at risk. |
|  | 15c | To promote adherence, intervention sessions will be scheduled in advance and integrated into participants’ treatment plans. Therapists will provide reminders and motivational support to encourage engagement. Attendance will be tracked, and missed sessions will prompt follow-up contact to identify barriers and reschedule. Fidelity will be monitored through regular supervision and periodic review of session notes. Additionally, participant feedback will be collected through brief check-ins to address concerns and maintain acceptability. |
|  | 15d | Participants will be permitted to continue receiving standard inpatient care, including medical management, supportive counseling, family therapy and pharmacological treatment as clinically indicated. Participation in routine ward activities and non-conflicting therapeutic groups will also be allowed. However, the introduction of other structured psychotherapeutic interventions targeting the same primary outcomes—such as trauma-focused therapy outside the study protocol—will not be permitted during the trial period to prevent contamination and confounding effects. |
| Outcomes | 16 | Manuscript: Methods - Design  Table 1, Attachment 1,  Methods - Measurements. |
| Harms | 17 | Manuscript: Methods - Assessment and management of harmful behavior.  Methods – measurement – Safety - Aversive behaviours: Self-harm, Suicide behaviour |
| Participant timeline | 18 | Time schedule of enrolment, interventions (including any run-ins and washouts), assessments, and visits for participants. A schematic diagram is highly recommended (see fig 1)  Manuscript. Figure 2 |
| Sample size | 19 | Manuscript: Methods – Design – Recruitment of participants - sample size. |
| Recruitment | 20 | Strategies for achieving adequate participant enrolment to reach target sample size.  Manuscript: Methods - Design - recruitment of participants, Figure 1  Recruitment Capacity: The three-year recruitment period aligns with institutional capacity (annual turnover of 20–30 patients), making it feasible to reach the target sample of approximately 75 participants from an estimated 80 invitations. |
| **Methods: Assignment of interventions** | | |
| Randomisation: |  | Not relevant as this is a non RCT single arm study, |
| Sequence generation | 21a | Not relevant |
|  | 21b | Not relevant |
| Allocation concealment mechanism | 22 | Not relevant |
| Implementation | 23 | Not relevant |
| Blinding | 24a | Not relevant |
|  | 24b | Not relevant |
|  | 24c | Not relevant |
| **Methods: Data collection, management, and analysis** | | |
| Data collection methods | 25a | Manuscript: Methods - Data protection, management and storage |
|  | 25b | All participants who drop out from the treatment intervention (DBT. SUD skills training and/or NET) but completing the standard treatment at the treatment center will be a part of the follow-up data collection. Dropout from the standard treatment will mean dropout from the study, and those will not be a part of the follow up data collection. |
| Data management | 26 | Manuscript: Methods - Data protection, management and storage. |
| Statistical methods | 27a | Manuscript: Methods – Statistical analysis |
|  | 27b | All participants will be included in the analysis. |
|  | 27c | Missing data will not be imputed, as the study is designed as a feasibility trial rather than a hypothesis-testing study. The number and reasons for missing data will be documented and reported in accordance with CONSORT guideline |
|  | 27d | Not relevant |
| **Methods: Monitoring** | | |
| Data monitoring committee | 28a | A formal Data Monitoring Committee (DMC) will not be established for this feasibility study. The decision is based on a documented risk assessment showing that the intervention and procedures pose minimal risk to participants and that the study has a primary focus on feasibility rather than efficacy. Oversight of participant safety and trial conduct will be managed by the Research Committee and the clinic team according to predefined criteria (Manuscript: Method Table 1). |
|  | 28b | Interim analysis will be done every 6 months to ensure patient safety. The predefined stopping guidelines are as presented in Table 2 in the manuscript. The trial will proceed until all participants complete the intervention unless serious adverse events occur that compromise participant safety. In such cases, the decision to terminate or modify the trial will be made collectively by the research committee in consultation with the trial sponsor following the risk management of harmful behavior presented in the manuscript  Manuscript: Methods - Assessment and management of harmful behavior. |
| Trial monitoring | 29 | Formal external monitoring is not implemented because this is a single-arm feasibility study with low anticipated risk and no investigational medicinal products. Instead, trial conduct is overseen internally by the research team and the trial sponsor through routine supervision meetings and adherence checks. These include verification of protocol compliance, data integrity, and participant safety during scheduled team consultations. Any protocol deviations or adverse events are documented and reviewed promptly. The absence of a dedicated monitoring committee is justified by the limited scope, non-randomized design, and feasibility focus of the study. |
| **Ethics** | | |
| Research ethics approval | 30 | The project has approval from both the Regional Ethics Committee (REC) and Data Protection Services (DPS) attained #203 428/2020**.** |
| Protocol amendments | 31 | All major changes to the protocol must be agreed to by the research committee and regional ethic committee. Relevant parties such as clinical staff and participants will be informed either by email or by letter. |
| Consent or assent | 32a | With informed consent form.  [Study Details \| NCT06345053 \| Presence of PTSD and Emotion Dysregulation Among Inpatients With Substance Use Disorder \| ClinicalTrials.gov](https://clinicaltrials.gov/study/NCT06345053?cond=PTSD&term=Substance%20Use%20Disorder&intr=DBT&rank=2#more-information) |
|  | 32b | Not applicable |
| Confidentiality | 33 | Manuscript: Methods - Data protection, management and storage. |
| Ancillary and post-trial care | 34 | Potential harm is assessed as low. In case of increased symptoms by the data collection or intervention, A therapist and other staff at the clinic will be there to help the patient regulate possible transitory increase in PTSD symptoms. A therapist and other staff at the clinic will be there to help the patient. Following up after completed treatment will be standard clinical care for discharges patients from the clinic. |

SPIRIT=Standard Protocol Items: Recommendations for Interventional Trials.
